# Social Isolation, Loneliness and Well-Being: The Impact of WeChat Use Intensity During the COVID-19 Pandemic in China

**DOI:** 10.3389/fpsyg.2021.707667

**Published:** 2021-08-10

**Authors:** Jianfeng Li, Luyang Zhou, Beatrice Van Der Heijden, Shengxiao Li, Hong Tao, Zhiwen Guo

**Affiliations:** ^1^Department of Big Data Management and Application, School of Business, Hubei University, Wuhan, China; ^2^Department of Business Administration, School of Business, Shaoxing University, Shaoxing, China; ^3^Institute for Management Research, Radboud University, Nijmegen, Netherlands; ^4^Faculty of Management, Open University of the Netherlands, Heerlen, Netherlands; ^5^Department of Marketing, Innovation and Organisation, Ghent University, Ghent, Belgium; ^6^School of Business, Hubei University, Wuhan, China; ^7^Kingston Business School, Kingston University, London, United Kingdom; ^8^Department of Human Resource Management, School of Business, Hubei University, Wuhan, China

**Keywords:** lockdown social isolation, lockdown loneliness, well-being (lockdown stress and lockdown life satisfaction), lockdown WeChat use intensity, COVID-19

## Abstract

This study is aimed to examine the impact of WeChat use intensity on social isolation, loneliness, and well-being during the lockdown period of the COVID-19 pandemic. Drawing on the regulatory loop model of loneliness, the notions of Internet Paradox, the Time Displacement hypothesis and previous literature on WeChat use intensity, we propose that lockdown loneliness (partially) mediates the relationship between lockdown WeChat use intensity and well-being (i.e., lockdown stress and lockdown life satisfaction). Moreover, we assume that lockdown WeChat use intensity moderates the relationship between lockdown social isolation and well-being (i.e., lockdown stress and lockdown life satisfaction) in both a direct and in an indirect way, that is through lockdown loneliness. The results from our Structural Equation Modeling analyses, using a sample of 1,805 Chinese respondents, indicate that all of our research hypotheses are confirmed. From this empirical work, it becomes clear that online social interactions, which are believed by many people to be able to compensate for the lack of offline social interactions during the COVID-19 lockdown period, in fact are endangering their mental health and life satisfaction instead. This article concludes with theoretical and practical implications of our study, followed by its limitations and recommendations for future research.

## Introduction

The COVID-19 (COrona VIrus Disease) pandemic that broke out in December 2019 is an unprecedented global public health crisis, as of 27 June 2021, more than 180 million people worldwide are or have been diagnosed with the virus, and with already more than 3.9 million deaths as a result (World Health Organization (WHO), 2021). In response to the COVID-19 pandemic, many governments implemented measures, such as the lockdown and social distancing regulations, to prevent the spreading of the virus and to cope with the extreme burden that is put on the healthcare system. This lockdown and social distancing regulations force people to isolate themselves at home and to cut off face-to-face gatherings and interactions within family members, friends, colleagues, classmates, and so on. However, as human beings are in essence social animals, social relationships are among the most fundamental elements in their lives. Therefore, in the COVID-19 pandemic, social media (e.g., Facebook and WeChat) has been widely used among people to keep close social connections with others and to help them to cope with the various demands in their work and private lives, that had to be combined at home with all of its accompanying challenges.

Based on the data from Tencent (https://www.tencent.com/zh-cn/investors.html), published on May 20th 2021, WeChat (WeiXin in Chinese), as a popular social media tool, has become an important part of Chinese people's daily work and life, with more than 1.2 billion active users worldwide, on a monthly basis. Chinese people not only use WeChat app to conduct commercial transaction, to work, and to learn, but also use WeChat app to shop, to play games, and to communicate with each other. In addition, WeChat has also been widely used for prevention of infection with the COVID-19 virus and for close surveillance in China. For example, during the period of the COVID-19 pandemic, Chinese people had to submit their health reports, using the WeChat app, daily; and they needed to scan a health code using the WeChat app to enter and leave public places. As a result of this, WeChat use is like a double-edged sword. WeChat use can not only help an individual to enhance interpersonal interactions and social connections, and, through this, to improve their subjective well-being (He and Huang, 2020), but it may also increase one's loneliness due to the lack of face-to-face interpersonal interactions (Jiao, 2016). At the same time, through WeChat groups, professionals could provide professional consultation and psychological counseling services for home-quarantined people in the COVID-19 pandemic (Hu et al., 2020).

From recent research, we already know that social isolation is an important predictor of loneliness (Cheng et al., 2020; Tomova et al., 2020), that both social isolation and loneliness have detrimental effects on one's well-being (Hwang et al., 2020; Lewis et al., 2020; Kasar and Karaman, 2021), and that social media use may have both positive and negative impacts on one's well-being in the COVID-19 pandemic (Gonzlez-Padilla and Tortolero-Blanco, 2021). Additionally, in China, some researchers found that Internet use can significantly improve individuals' subjective well-being (Zhou and Sun, 2017; Zhu and Leng, 2018), while others reported that frequency of Internet use did significantly enhance subjective well-being (Long and Yi, 2019). Accordingly, we posit that WeChat use intensity may be an important determinant of these variables as well.

To the best of our knowledge, so far, no empirical studies that dealt with the relationships between WeChat use intensity, social isolation, loneliness, and well-being have been performed, let alone scholarly research that focused on Chinese self-quarantined residents during the COVID-19 pandemic. Therefore, the current study aims to investigate whether lockdown loneliness (partially) mediates the relationship between WeChat use intensity and well-being (i.e., lockdown stress and lockdown life satisfaction) within a Chinese context. Moreover, we also examine whether WeChat use intensity moderates the relationship between social isolation and well-being (i.e., lockdown stress and lockdown life satisfaction), both in a direct way and in an indirect way, that is through lockdown loneliness. The findings in this study will help us to better understand the influence mechanism of WeChat use intensity on people's well-being during the COVID-19 pandemic, and to provide evidence-based recommendations for sufferers on how to manage their use of WeChat in a pandemic situation.

## Theoretical Background and Hypotheses

### Social Isolation, Loneliness, and Well-Being

Social isolation usually refers to a paucity of social relationships or social connections (Tanskanen and Anttila, 2016; Kobayashi and Steptoe, 2018), while loneliness refers to the subjective experience of social isolation (Hawkley and Cacioppo, 2010; Kobayashi and Steptoe, 2018). The emotional state of loneliness has often been viewed as biological concomitants or responses of social isolation (Steptoe et al., 2013), and people usually feel lonely and crave social contacts after acute social isolation, just like the way fasting causes hunger (Tomova et al., 2020). However, as Kiyoshi (1987)) noted, “Loneliness is not in the mountains but in the streets, not in one person but among many people” (p. 59), yet, social isolation and loneliness do not necessarily occur at the same time, or one after the other. What we know for sure, is that both social isolation and loneliness have negative effects on well-being, and that such detrimental effects can be interpreted by the stress-buffering and main effects models of social relationships (Holt-Lunstad et al., 2010), and by the regulatory loop model of loneliness (Hawkley and Cacioppo, 2010).

Social relationships are central to well-being as human beings live in groups. In particular, first, the stress-buffering model suggests that social relationships may provide social support (emotional, instrumental, or informational) that improves neuroendocrine or adaptive behavioral responses to acute or chronic stressors (e.g., self-quarantine, social transitions) (Cohen, 2004). Social support thereby buffers or moderates the harmful influence of stressors on well-being. Accordingly, self-quarantined people might suffer more from stress in comparison with others, due to a lack of social connectedness, which, in turn, might bring about an adverse effect on their well-being.

Second, the main effect model emphasizes that social connectedness is beneficial for well-being, irrespective of whether individuals are under stress or not, and social connectedness may be linked to protective health effects by means of cognitive, emotional, behavioral, and biological influences (Cohen, 2004). For example, being a member of a badminton club may give individuals more opportunities to exercise. Analogously, when people feel lonely due to self-quarantine, they might relieve stress, keep healthy and protect their life satisfaction by exercising at home. Recent research also confirmed that people who do exercise almost every day during the COVID-19 pandemic period usually have the best mood (Brand et al., 2020). Indeed, we have managed to see some videos through WeChat groups, wherein Chinese people play badminton during the self-quarantine period.

Third, the regulatory loop model of loneliness (Hawkley and Cacioppo, 2010) proposes that social isolation is divided into objective social isolation and perceived social isolation. Loneliness is synonymous with perceived social isolation and refers to “a distressing feeling that accompanies the perception that one's social needs are not being met by the quantity or especially the quality of one's social relationships” (p. 218). Loneliness is also regarded as feeling unsafe, and this sense of unsafety may trigger implicit hypervigilance for social threat in one's surroundings. Unconscious surveillance for social threats may lead to cognitive biases, namely, people who feel lonely or isolated see society as a more threatening place than those who are not alone, anticipate more negative social interactions, and remember more negative social messages.

In other words, lonely people's hypervigilance for society can result in more negative feelings and attitudes toward others; and they hunger for establishing a sense of psychological security and protection once they feel that their interpersonal needs are not met, and that specific situations make them feel lonely or isolated (Hawkley and Cacioppo, 2010). Doing so makes lonely people even more alert and sensitive to social relationships, and prompts them to continually assess the situation and judge whether their interpersonal relationships meet the need for belonging or not. In general, loneliness can produce a negative loop of social interactions; and the stronger the loneliness of a person is, the more likely he or she develops negative attributions to others, which, in turn, lead to negative behaviors and a decrease in belonging and security (Hawkley and Cacioppo, 2010). Correspondingly, this loop of self-reinforcing loneliness, which we expect to have occurred during the COVID-19 pandemic, is likely to be accompanied by feelings of stress (anxiety) and life dissatisfaction.

In addition, Cornwell and Waite's (2009a) found that social isolation could exert an influence on self-rated physical health and mental health through a mediation effect of loneliness. Well-being could be defined as a multi-dimensional construct that includes the absence of negative affects, such as stress, and the presence of positive affects, such as life satisfaction (Diener, 1984; Houben et al., 2015; Utz and Breuer, 2017). Mental well-being could be defined as a positive state of psychological and emotional health, in which one can cope with the normal stressors of life (Tuzovic and Kabadayi, 2020). Therefore, based upon the theoretical outline given above, we formulated the following hypotheses:

***Hypothesis 1a****: Lockdown social isolation is positively associated with lockdown loneliness in the COVID-19 lockdown period*.
***Hypothesis 1b****: Lockdown loneliness (partially) mediates the relationship between lockdown social isolation and lockdown stress in the COVID-19 lockdown period*.
***Hypothesis 1c****: Lockdown loneliness (partially) mediates the relationship between lockdown social isolation and lockdown life satisfaction in the COVID-19 lockdown period*.

### The Influence of WeChat Use Intensity on the Relationships Between Social Isolation, Loneliness and Well-Being

Social media refers to “a group of Internet-based applications that build on the ideological and technological foundations of Web 2.0, and that allow the creation and exchange of User Generated Content” (Kaplan and Haenlein, 2010, p. 61). Social media use can be defined as various activities performed by human beings through social media platforms, such as providing a personal profile for self-presentation, online interpersonal interactions, and a stream of frequently updated content (e.g., WeChat's News Feed) (Verduyn's et al., 2017). The measurement of social media use is generally divided into two main categories: intensity and addiction, which evaluate how much time (frequency or intensity) an individual spends on social media or experiences feelings of connectedness to the platforms, as well as assess an individual's addictive behaviors or symptoms related to social media (Mieczkowski et al., 2020). In our empirical study, intensity is chosen to assess the impact of social media use (i.e., WeChat use intensity).

At present, social media in China mainly includes online interactive modes such as WeChat, QQ, Weibo, Blog, Forum, Podcast and social networking sites, which provide an ideal impression management platform and more control over social interactions for individuals. WeChat is China's most popular messaging app that integrates instant communications, social contacts, e-commerce, mobile payments, and public services. Chinese people have incorporated WeChat into their daily lives by using it to communicate with family and friends, to shop, to play games, and to pass time. Whether social use of WeChat has positive or negative effects depends on whether WeChat enables an individual to keep a sound balance between close and loose social connections that they establish and maintain. As far as interpersonal relationships are concerned, online relationships are usually a representation and extension of offline relationships. That is to say, in case the offline relationships are close, the online relationships are also close; and vice versa. For example, family space, friend space (e.g., Moments), colleague space, and classmate space are reflections of such close or loose interpersonal relationships.

#### WeChat Use Intensity, Loneliness, and Well-Being

As social media use intensity usually refers to the frequency of social media use (Boer et al., 2021), in this contribution WeChat use intensity refers to the frequency of WeChat use or feelings of connectedness to WeChat. The relationships between WeChat use intensity, loneliness, and well-being can be explained by the notion of Internet Paradox (Kraut and Burke, 2015) and the hypothesis of Time Displacement (Putnam, 2000). First, unlike empirical studies that concluded that increased use of the Internet was related to reduced stress, depression, and loneliness (Shaw and Gant, 2002; Teppers et al., 2014), because individuals would have more chances to receive social support from online interactions, Kraut and Burke (2015) contended that an increased use of the Internet could decrease the amount of social support because of the weaker social ties in online settings. As a result of the diminished social support, individuals may experience more stress, loneliness, and life dissatisfaction. More specifically, strong social ties (e.g., communications with family members and close friends online) may have positive effects on life satisfaction and negative effects on loneliness and stress. On the contrary, weak social ties (e.g., communications with strangers) may have harmful effects on psychological well-being (Kraut and Burke, 2015).

Second, the hypothesis of Time Displacement (Putnam, 2000) suggests that individuals' time is limited and that the amount of time spent on the Internet may crowd out time spent in socializing (Putnam, 2000). Consequently, the time for face-to-face communications will reduce with a higher amount of Internet use, loneliness will increase and psychological well-being will decrease. In a similar vein, Turkle (2014) stated that interpersonal relationships will be reduced into simple relations in case the Internet technology is utilized to deal with intimate relationships; continuous online communications, in turn, will lead to anxiety concerning losing one's contacts; and whereas it makes them connect more closely, the Internet use will make individuals more isolated.

As far as empirical work is concerned, recent research has demonstrated that excessive use of social media was positively associated with loneliness and anxiety (Boursier et al., 2020), and that WeChat use could exert a negative impact on social interaction behaviors (Xu et al., 2020), as offline social relationships are critical to human well-being. Based on the outline given above, and in combination with Hypothesis 1b-1c, we formulated the following hypotheses:

***Hypothesis 2a****: Lockdown WeChat use intensity is positively associated with lockdown loneliness in the COVID-19 lockdown period*.
***Hypothesis 2b****: Lockdown loneliness (partially) mediates the relationship between lockdown WeChat use intensity and lockdown stress in the COVID-19 lockdown period*.
***Hypothesis 2c****: Lockdown loneliness (partially) mediates the relationship between lockdown WeChat use intensity and lockdown life satisfaction in the COVID-19 lockdown period*.

#### WeChat Use Intensity as a Moderator in the Relationship Between Social Isolation and Loneliness

In order to better understand the impact of WeChat use intensity on social isolation and loneliness, we build upon the notion of Internet paradox (Kraut et al., 1998). Kraut et al. (1998), in their two-year longitudinal study, reported that people used the Internet mainly for interpersonal communication; that the Internet reduced the importance of face-to-face communications in creating and maintaining strong social ties; and that the more time they spent in using the Internet, the stronger they felt depression and loneliness. On the contrary, Shaw and Gant (2002) found that Internet use decreased loneliness and depression because, online, individuals had more opportunities to meet and form friendships with others, which made them feel less lonely. From the longitudinal study by Teppers et al. (2014), it appeared that, while using Facebook to make new friends reduced loneliness, with the passage of time, using Facebook to compensate for social skills increased loneliness.

Furthermore, some scholars found that the more time young adults spent in using social media, the greater they felt dispositional anxiety (Vannuccia et al., 2017). While WeChat use may improve interpersonal interactions and social connections (He and Huang, 2020), excessive use of WeChat may increase loneliness due to a lack of face-to-face social communications (Jiao, 2016). In addition, Kraut and Burke (2015) posited that whether Internet use has positive or negative effects depends on how individuals use the Internet, what they talk about, and whom they talk to.

Apparently, during the COVID-19 pandemic, individuals spend more time in the Internet use and WeChat use than common because social isolation (e.g., self-quarantine) cuts off face-to-face communications, and online social contacts substitute offline communications. We assume that individuals use the Internet and WeChat more frequently or excessively, and in combination with Hypothesis 1a, we formulated the following:

***Hypothesis 3a****: Lockdown WeChat use intensity positively moderates the relationship between lockdown social isolation and lockdown loneliness in the COVID-19 lockdown period*.
***Hypothesis 3b****: Lockdown WeChat use intensity positively moderates the relationship between lockdown social isolation and lockdown stress, through lockdown loneliness, in the COVID-19 lockdown period*.
***Hypothesis 3c****: Lockdown WeChat use intensity negatively moderates the relationship between lockdown social isolation and lockdown life satisfaction, through lockdown loneliness, in the COVID-19 lockdown period*.

**Figure 1:**
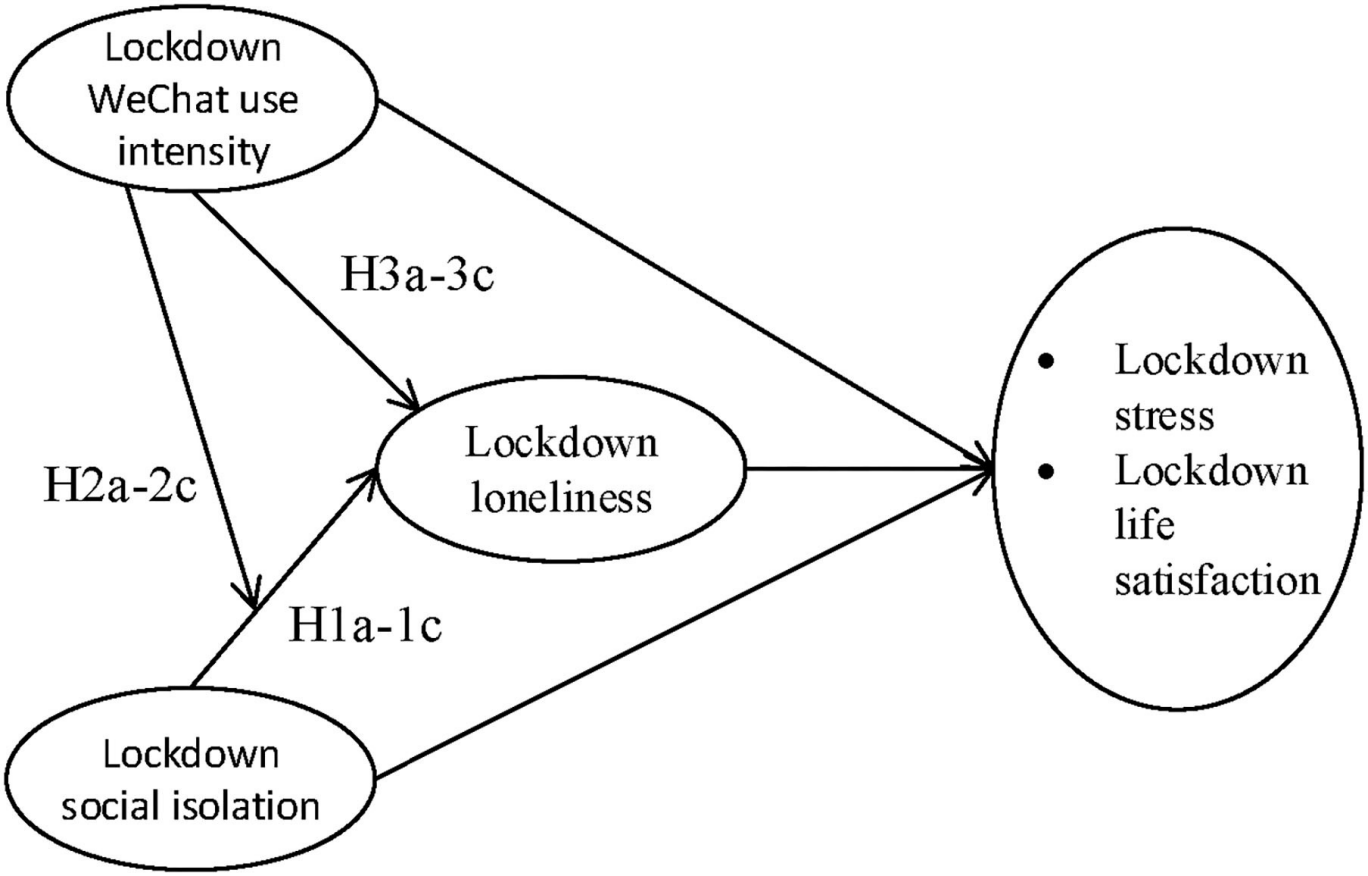
Conceptual model.

Our study model is depicted in Figure 1.

## Method

### Sample and Procedure

A pilot study was conducted among acquaintances by using convenience sampling (Sedgwick, 2013) in July, 2020. Three hundred and nineteen valid questionnaires were collected during an on-line survey, and were used to revise the pilot questionnaire into the final version. The main survey study was conducted by using stratified sampling (Thompson, 2012) in August 2020. More specifically, first, based on the data of “Baidu's Epidemic Real-time Big Data Report” published on July 29, 2020, and on China's demographic data from 2019, we divided China into three regions for our sampling strategy. The first region was Hubei Province, with a resident population of 4.2 per cent of China's total, where 68,135 people were infected with COVID-19; the second region included six provinces (Guangdong, Henan, Zhejiang, Hunan, Anhui and Heilongjiang), with a resident population of 31.5 per cent of China's total, where 7,177 people were infected with COVID-19; and the third region included other provinces, with a resident population of 64.3 per cent of China's total, where 12,368 people were infected with COVID-19.

Second, in order to obtain a suitable amount of data for Structural Equation Modeling (SEM), following our stratified sampling approach, we strived to collect 2,000 valid surveys including 400, 800, and 800 respondents in the first, second and third regions, respectively.

Third, online questionnaires were collected through the following three ways. First, WeChat groups were used to collect data in the first region. Here, all participants completed the survey voluntarily and anonymously, and received a certain amount of cash (i.e., 8.88, 11.0 and 23.6 CNY) as compensation (*N* = 893). Second, we signed a sample service contract with Questionnaires Platform, through which data was randomly collected across the nationwide, and 644 completed questionnaires were obtained. Third, we signed a sample service contract with the NetEase positioning platform, through which the data was randomly collected across China, and 620 completed questionnaires were collected. To sum up, 2,157 completed questionnaires were obtained.

Next, the filled out questionnaires were validated. In particular, those respondents who had not experienced the lockdown or other enclosed anti-epidemic modes, those who had no work units during the period of the lockdown or other enclosed management, those who had retired or worked in rural areas, and those who lived in Hong Kong, Macao and Taiwan or abroad were eliminated from the final data set. Finally, 1,805 valid questionnaires were gathered including 358, 745, and 702 in the first, second and third regions, respectively.

Table 1 shows the social-demographic characteristics of the sample.

**Table 1:** The socio-demographic characteristics of the sample.

**Description of Variable**	***N* (%)**
**Gender** female male	812(45%) 993(55%)
**Age** born in/after 2000 (at least 18 years old) born in 1990s born in 1980s born in 1970s born in 1960s born in 1950s or earlier	7(0.4%) 557(30.9%) 839(46.5%) 288(16.0%) 111(6.1%) 3(0.2%)
**Level of education** junior high school graduate or below senior high school graduate college degree bachelor's degree Master's degree PhD.	11(0.6%) 79(4.4%) 298(16.5%) 1050(58.2%) 279(15.5%) 88(4.9%)
**Income**	
[How much on average did you earn monthly last year (CNY)?]	
< = 999 1,000–2,999 3,000–4,999 5,000–9,999 10,000–19,999 20,000–49,9999 50,000–99,999 > 100,000	4(0.2%) 46(2.5%) 283(15.7%) 797(44.2%) 472(26.1%) 116(6.4%) 50(2.8%) 37(2.0%)

### Measures

*Lockdown social isolation*. Considering the context of the COVID-19 lockdown, the measure for social isolation focused on external aspects (Zavaleta et al., 2017), such as infrequent contact with network members (Brummett et al., 2001), low participation in social activities (Ellison and George, 1994; Thoits and Hewitt, 2001; Benjamins, 2004), and social disconnectedness (Cornwell and Waite, 2009b). Lockdown social isolation was assessed using a four-item (α = 0.83) scale (e.g., “I hadn't seen many of my family members whom I should have seen if there had been no lockdown?”). The response categories ranged from 1 (“strongly disagree”) to 5 (“strongly agree”).

*Lockdown loneliness*. Loneliness can be divided into emotional loneliness and social loneliness; emotional loneliness stems from the lack of “family attachment,” while social loneliness stems from the lack of “social overall relationship” (Gierveld and Van Tilburg, 2006). Given the fact that individuals usually lived with their families in the Spring Festival, there was no lack of “family attachment” during the enclosed management of the COVID-19 pandemic in China. Accordingly, only social loneliness was incorporated in this study. Lockdown loneliness was assessed using a five-item (α = 0.88) based on the scales by Hughes et al. (2004) and Schrempft et al. (2019) that measure lockdown social loneliness (e.g., “During the COVID-19 lockdown, I often felt that I lacked companionship”). The response categories ranged from 1 (“strongly disagree”) to 5 (“strongly agree”).

*Well-being*. Based on the research by Diener (1984) and Utz and Breuer (2017), in this study, well-being was operationalized as stress and life satisfaction. *Lockdown stress* was assessed using a five-item scale (α = 0.92) based on the scales by Antony et al. (1998) and Lee et al. (2019) that measure stress (e.g., “During the period of COVID-19 enclosed management, I remember that I often felt nervous and anxious at that time”). The response categories ranged from 1 (“strongly disagree”) to 5 (“strongly agree”).

*Lockdown life satisfaction* was assessed using a five-item scale (α = 0.82) based on the scales by Diener et al. (1985) and Margolis et al. (2019) that measure life satisfaction (e.g., “During the period of COVID-19 enclosed management, on the whole, I was satisfied with my life at that time”). The response categories ranged from 1 (“strongly disagree”) to 5 (“strongly agree”).

*Lockdown WeChat use intensity*. Given that in China, frequency of Internet use, rather than Internet use, appears to significantly increase subjective well-being (Long and Yi, 2019), in this study, we measured lockdown WeChat use intensity using a four-item scale (α = 0.88) based on scales developed by Moqbel et al. (2013), Ellison et al. (2007) and Wang et al. (2019). Sample items included: “During the period of COVID-19 enclosed management, I felt out of touch when I had not logged on to WeChat for a while.” The response categories range from 1 (“strongly disagree”) to 5 (“strongly agree”).

The details for all measures are presented in Appendix 1.

### Control Variables

Control variables included the experience of the lockdown, working status, family situation, gender, age, educational level, and income. The experience of the full lockdown, which means “the strictly and fully enclosed isolation of the whole city (or the whole region),” was coded as 1 while “less strict lockdown measures or partial lockdown” was coded as 0. Concerning working status, the respondents were divided into two categories: individuals who have stopped working due to epidemic prevention and control were coded as 1; while 0 referred to those individuals who worked in their unit or at home online. Family situation was coded as 1 in case individual lived together with family members, and it was coded 0 in case one lived alone.

In addition, we followed Fair (1978) for the data coding. Gender, 0 = female, 1 = male; Age, 19 = under 20, 25.5 = 20–30, 35.5 = 30–40, 45.5 = 40–50, 55.5 = 50–60, 65.5 = 60 or over. Educational level, 9 = grade school; 12= high school graduate or below; 15 = junior college graduate; 16 = college graduate; 18 = Master's degree, 20 = Ph.D., or other advanced degree; average month income, 0.05 = 1000 CNY or below, 0.2 = 1000–3000 CNY, 0.4 = 3000–5000 CNY, 0.75 = 5000–10,000 CNY, 1.5 = 10,000–20,000 CNY, 3.5 = 20,000–50,000 CNY, 7.5 = 50,000–100,000 CNY, 15 = over 100,000 CNY.

### Data Analysis

We followed Hair et al.'s recommendations Hair et al. (2010) to examine the discriminant validity of our measurement model, using SEM, and the following six model fit indices were computed: normed chi-square statistic (*x*^2^/df), goodness-of-fit index (GFI), Tucker-Lewis index (TLI), comparative fit index (CFI), root mean square error of approximation (RMSEA), and standardized root mean square residual (SRMR). As a rule of thumb, the *x*^2^/df ≤ 3 (Hair et al., 2010), GFI, TLI, and CFI values >0.90 (Bentler, 1990), a RMSEA ≤ 0.05 (Kenny et al., 2015), and a SRMR ≤ 0.08 (Hu and Bentler, 1999) indicate a close fit between the model and the data.

Subsequently, we conducted a series of confirmatory factor analyses to investigate whether all the variables that were examined in this study were distinct. Compared to other models (see Table 2), the proposed five-factor structure (i.e., lockdown social isolation, lockdown social loneliness, lockdown stress, lockdown life satisfaction, and lockdown WeChat use intensity) was found to be a significantly better fit with the data, *x*^2^/df = 580.562/125 = 4.64, *p* <0.001, CFI = 0.976, TLI = 0.970, RMSEA = 0.045, SRMR = 0.032. This finding suggested that all study variables were distinct from one another.

**Table 2:** Testing the discriminant validity of the constructs.

**CFA models**	***x*^**2**^**	***df***	***x*^**2**^/*df***	**CFI**	**TLI**	**RMSEA**	**SRMR**
5 factors	580.562	125.000	4.644	0.976	0.970	0.045	0.032
4 factors	2520.937	129.000	19.542	0.872	0.848	0.101	0.074
3 factors	4499.065	132.000	34.084	0.766	0.729	0.135	0.111
2 factors	7512.185	134.000	56.061	0.549	0.140	0.175	0.605
1 factor	9620.501	135.000	71.263	0.492	0.425	0.197	0.149

*5 factors model: lockdown stress, lockdown life satisfaction, lockdown social loneliness, lockdown WeChat use intensity, lockdown social isolation*.

*4 factors model: lockdown stress + lockdown life satisfaction, lockdown social loneliness, lockdown WeChat use intensity, lockdown social isolation*.

*3 factors model: lockdown stress + lockdown life satisfaction, lockdown social loneliness, lockdown WeChat use intensity + lockdown social isolation*.

*2 factors model: lockdown stress + lockdown life satisfaction + lockdown social loneliness, lockdown WeChat use intensity + lockdown social isolation*.

*1 factor model: lockdown stress + lockdown life satisfaction + lockdown social loneliness + lockdown WeChat use intensity + lockdown social isolation*.

We also followed Podsakoff et al.'s suggestions Podsakoff et al. (2003) to overcome the concern of common-method variance (CMV). First, to optimize the psychometric qualities of the measurements that were used in this study, we used well-validated scales. Second, we made sure that all participants complete the questionnaires anonymously. Furthermore, we conducted Harman's single-factor test to examine the CMV; it comprises a type of confirmatory factor analysis in which all the variables are specified to load onto one common factor (cf. Mossholder et al., 1998). The one-factor model appeared to have a very poor fit with the data, *x*^2^*/df* = 9620.501/135 = 71.26, *p* <0.001, CFI = 0.492, TLI = 0.425, RMSEA = 0.197, SRMR = 0.149. This indicated that a majority of the variance in our model was not explained by one single factor.

## Results

### Descriptive Statistics and Correlation Analysis

Means, standard deviations, and correlation coefficients for all the study variables are presented in Table 3. Lockdown social isolation had a significant positive correlation with lockdown social loneliness (*r* = 0.15, *p* <0.001), lockdown stress (*r* = 0.21, *p* <0.001) and lockdown WeChat use intensity (*r* = 0.20, *p* <0.001), whereas it had a significant negative correlation with lockdown life satisfaction (*r* = −0.06, *p* <0.05). Lockdown social loneliness appeared to have a significant positive correlation with lockdown stress (*r* = 0.55, *p* <0.001), while it had a significant negative correlation with lockdown life satisfaction (*r* = −0.27, *p* <0.001). Lockdown stress had a significant negative correlation with lockdown life satisfaction (*r* = −0.35, *p* <0.001). Moreover, lockdown WeChat use intensity had a significant positive correlation with lockdown social loneliness (*r* = 0.16, *p* <0.001) and lockdown stress (*r* = 0.21, *p* <0.001).

**Table 3:** Mean, standard deviations, and correlations matrix for the whole sample.

	**Mean**	**SD**	**Range**	**1**	**2**	**3**	**4**	**5**	**6**	**7**	**8**	**9**	**10**	**11**	**12**
1 Lockdown social isolation	3.53	0.91	1–5	1											
2 Lockdown social loneliness	2.34	0.96	1–5	0.148^***^	1										
3 Lockdown stress	2.79	1.06	1–5	0.209^***^	0.545^***^	1									
4 Lockdown life satisfaction	3.37	0.81	1–5	−0.059^*^	−0.274^***^	−0.349^***^	1								
5 Lockdown WeChat use intensity	3.85	0.87	1–5	0.196^***^	0.155^***^	0.208^***^	0.019	1							
6 Experience of the lockdown	0.32	0.46	0–1	0.131^***^	0.089^***^	0.156^***^	−0.092^***^	0.035	1						
7 Working status	0.21	0.41	0–1	−0.011	0.071^**^	0.054^*^	−0.081^***^	−0.014	0.104^***^	1					
8 Family situation	0.92	0.26	0–1	−0.095^***^	−0.099^***^	0.014	0.038	0.053^*^	−0.091^***^	0.052^*^	1				
9 Gender	0.55	0.50	0–1	0.020	0.105^***^	−0.046^*^	−0.017	−0.052^*^	−0.042	0.002	−0.085^***^	1			
10 Age	35.23	8.60	19–65.5	−0.084^***^	−0.124^***^	−0.093^***^	0.031	−0.034	−0.053^*^	−0.028	0.103^***^	0.122^***^	1		
11 Educational level	16.17	1.72	9–21	0.072^**^	−0.097^***^	−0.025	0.098^***^	−0.080^**^	−0.028	−0.192^***^	−0.003	−0.066^**^	0.052^*^	1	
12 Income	1.53	2.34	0.05–15	−0.018	−0.039	−0.103^***^	0.049^*^	−0.034	−0.026	−0.025	−0.012	0.029	0.047^*^	0.102^**^	1

* *p < 0.05*,

** *p < 0.01*,

*** *p < 0.001*.

We also found that our control variables were related to lockdown social isolation, lockdown social loneliness, lockdown stress, lockdown life satisfaction, and lockdown WeChat use intensity, and the mean for family situation (*M* = 0.92) confirmed that most of participants stayed with families. With these outcomes, we found preliminary evidence for our research hypotheses.

### Hypotheses Testing

**Figure 2:**
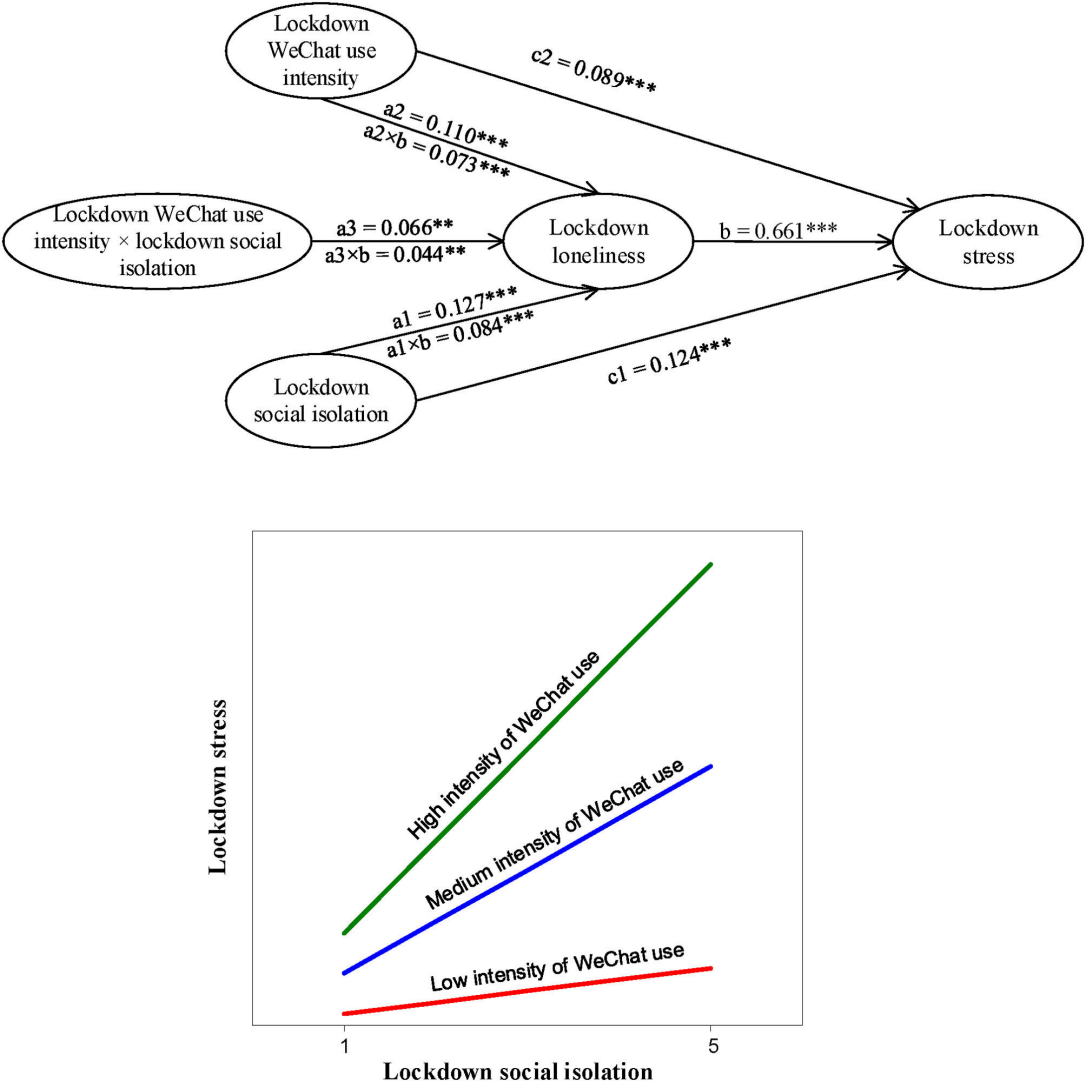
A graphical illustration of parameter estimates and moderation by intensity of WeChat use (lockdown stress as dependent variable). Number of Free Paramaters = 59, *LL* = −33208.532, *AIC* = 66535.063, **p <0.01, ***p <0.001, *n* = 1805. Note.—The indirect effect of Lockdown Social Isolation on Lockdown Stress is conditional on Intensity of WeChat Use: (a1 × b) + (a3 × b) × Lockdown WeChat use intensity.

**Figure 3:**
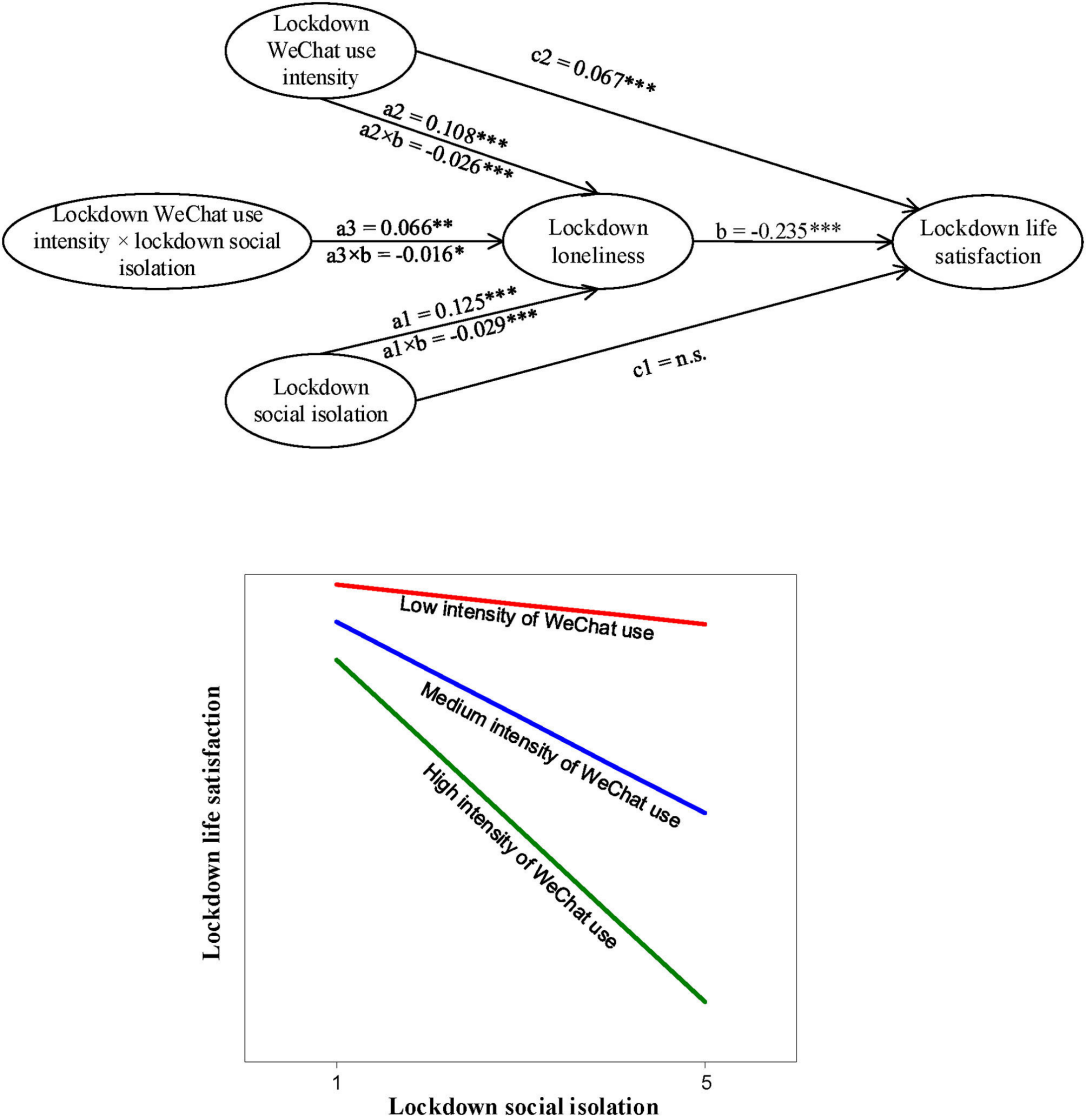
A graphical illustration of parameter estimates and moderation by intensity of WeChat use (lockdown life satisfaction as dependent variable). Number of Free Paramaters = 53, *LL* = −28308.964, *AIC* = 56723.929, *p <0.05, **p <0.01, ***p <0.001, n.s. not significant. *n* = 1805. Note.—The indirect effect of Lockdown Social Isolation on Lockdown Life Satisfaction is conditional on Intensity of WeChat Use: (a1 × b) + (a3 × b) × Lockdown WeChat use intensity.

We used latent moderated structural model (LMS) (Klein and Moosbrugger, 2000) within Mplus Version 8.3 to test the structural model as shown in Figures 2, 3, which incorporate all research hypotheses. First, we tested the model with lockdown stress as the dependent variable (see Figure 2). In particular, we started with estimating a model with the direct effect and the mediation effect only, i.e., excluding the interaction effect, which demonstrated a satisfactory overall model fit: *x*^2^*/df* = 883.672/182 = 4.855, *p* <0.001, CFI = 0.957, TLI = 0.951, RMSEA = 0.046, SRMR = 0.049. Next, we estimated the proposed model with the interaction term included, which appeared to significantly improve the model fit, −*2*Δ*LL* = 6.958, Δ*df* = 1, *p* <0.01 (the difference between the log-likelihood LL0 of the baseline model (M0) and the log-likelihood LL1 of the wherein the interaction term was added (M1) multiplied by −2, i.e. −*2*Δ*LL* = −2(LL0–LL1), is chi-square distributed, cf. Gerhard et al., 2015).

Second, analogously, we tested the model with lockdown life satisfaction as dependent variable (see Figure 3). We estimated the baseline model with the direct effect and the mediation effect only, i.e., excluding the interaction effect, which demonstrated a satisfactory overall model fit: *x*^2^*/df* = *x*^2^/df = 613.846/143 = 4.293, *p* <0.001, CFI = 0.960, TLI = 0.953, RMSEA = 0.043, SRMR = 0.047. We then estimated the proposed model with the interaction included. Adding the interaction term appeared to significantly improve the model fit: −2ΔLL = 6.888, Δ*df* = 1, p <0.01 (cf. Gerhard et al., 2015).

Hypotheses 1a, 1b, and 1c stated that in the COVID-19 lockdown period, lockdown social isolation is positively related to lockdown social loneliness, which, in turn, (partially) mediates the relationship between lockdown social isolation, on the one hand, and lockdown stress and lockdown life satisfaction, on the other hand. From Figures 2, 3, we can infer that after controlling for experience of the lockdown, working status, family situation, gender, age, educational level, and income, lockdown social isolation was significantly related to lockdown social loneliness (β = 0.13, *p* <0.001), which, in turn, partially mediated the relationship between lockdown social isolation and lockdown stress (β = 0.08, *p* <0.001), and fully mediated the relationship with lockdown life satisfaction (β = −0.03, *p* <0.001). With these outcomes, we have found full support for Hypotheses 1a, 1b, and 1c.

Hypotheses 2a, 2b and 2c stated that lockdown WeChat use intensity is positively related to lockdown social loneliness, which, in turn, (partially) mediates the relationships between lockdown WeChat use intensity, on the one hand, and lockdown stress and lockdown life satisfaction, on the other hand. Figures 2, 3 indicate that after controlling for experience of the lockdown, working status, family situation, gender, age, educational level, and income, lockdown WeChat use intensity was indeed positively related to lockdown social loneliness (β = 0.11, *p* <0.001), which, in turn, partially mediated the relationships between lockdown WeChat use intensity and lockdown stress (β = 0.07, *p* <0.001), and between lockdown WeChat use intensity and lockdown life satisfaction (β = −0.03, *p* <0.001). With these outcomes, we also found full support for Hypotheses 2a, 2b and 2c.

Hypotheses 3a, 3b and 3c predicted that lockdown WeChat use intensity positively moderates the relationship between lockdown social isolation and lockdown social loneliness, and further, that lockdown WeChat use intensity positively moderates the relationship between lockdown social isolation and lockdown stress, through lockdown social loneliness, and last, that lockdown WeChat use intensity negatively moderates the relationship between lockdown social isolation and lockdown life satisfaction, through lockdown social loneliness. Figures 2, 3 display that when the experience of the lockdown, working status, family situation, gender, age, educational level, and income were controlled for, lockdown WeChat use intensity indeed positively moderated the relationship between lockdown social isolation and lockdown social loneliness (β = 0.07, *p* <0.01). In addition, lockdown WeChat use intensity appeared to positively moderate the relationship between lockdown social isolation and lockdown stress, through lockdown social loneliness (β = 0.04, *p* <0.01), while lockdown WeChat use intensity appeared to negatively moderate the relationship between lockdown social isolation and lockdown life satisfaction, through lockdown social loneliness (β = −0.02, *p* <0.01). With these outcomes, we also found full support for Hypotheses 3a, 3b and 3c. The outcomes of the simple slopes' analyses results illustrated that the predicted positive relationship between lockdown social isolation and lockdown stress, through lockdown social loneliness, was stronger for self-quarantined individuals who reported a higher lockdown intensity of WeChat use in comparison with those individuals who reported a lower lockdown intensity of WeChat use (see Figure 2). In addition, it was found that the predicted negative relationship between lockdown social isolation and lockdown life satisfaction, through lockdown social loneliness, was stronger for self-quarantines individuals with a higher lockdown intensity of WeChat use in comparison with those with a lower lockdown intensity of WeChat use (see Figure 3).

## Discussion

### Reflections and Contributions

In order to explore the relationships between WeChat use intensity and social isolation, loneliness, and well-being, we tested a moderated mediation model using SEM, herewith building on the regulatory loop model of loneliness (Hawkley and Cacioppo, 2010), and the notions of Internet Paradox (Kraut and Burke, 2015), and the Time Displacement hypothesis (Putnam, 2000). To the best of our knowledge, this is the first empirical study using a nationwide Chinese sample to explain the impact of lockdown WeChat use intensity on lockdown social isolation, lockdown loneliness, and well-being (i.e., lockdown stress and lockdown life satisfaction) among self-quarantined people during the COVID-19 pandemic. Accordingly, this study contributes to the already existing literature in several ways.

First, unlike previous studies that indicated that loneliness did not exert a mediating effect in the relationships between social isolation, on the one hand, and physical health (e.g., chronic lung disease, arthritis) and well-being (e.g., depression symptoms, quality of life), on the other hand (Steptoe et al., 2013), we found that lockdown social isolation in a period of lockdown could affect well-being (i.e., lockdown stress and lockdown life satisfaction) through lockdown social loneliness. This finding is in line with the work by Cornwell and Waite's (2009a) who reported that loneliness could mediate the relationship between social isolation and self-rated physical health and mental health. Tuzovic and Kabadayi (2020) thought that mental well-being is a positive state of psychological and emotional health, in which one can cope with the normal stressors of life. Correspondingly, given the lack of research on the interrelatedness of social isolation, loneliness and health outcomes up to now (Mehrabia and Béland, 2020), our empirical findings extend previous scholarly research and, besides, add to the knowledge in this field by using a Chinese sample.

Second, by examining the role of WeChat use intensity as a predictor in the mediation model, with loneliness as a mediator, and well-being (i.e., stress and life satisfaction) as the outcome variable, we have explicitly addressed the impact mechanism through which WeChat use intensity effects individuals' well-being. In doing so, we have also extended previous scholarly work that focused on the influence of Internet use or social media use on loneliness and well-being. Building on the notion of the Internet Paradox (Kraut and Burke, 2015) and the Time Displacement hypothesis (Putnam, 2000), as the underlying frameworks of our study, we found that lockdown WeChat use intensity positively affects lockdown social loneliness, which, in turn (partially) mediates the effect of lockdown WeChat use intensity, on the one hand, and lockdown stress and lockdown life satisfaction, on the other hand. More specifically, lockdown WeChat use intensity can increase lockdown stress and decrease lockdown life satisfaction in a direct way, and in an indirect way, that is through lockdown social loneliness, as well.

Recent scholarly work illustrated that social media use had both positive and negative effects on one's well-being during the period of the COVID-19 isolation (Gonzlez-Padilla and Tortolero-Blanco, 2021). In addition, Kantar Group (2018) also found that for Chinese people, social media use (e.g., WeChat use, Weibo) could relieve their stress and improve their life experience (e.g., interpersonal communications with families and friends, online shopping, and self-presentation), whereas, at the same time, it could increase their stress (anxiety) (e.g., less sleep, poor eyesight, and privacy security) due to spending more time in maintaining interactions with others online. In accordance with these findings, our study reveals that WeChat use intensity is a double-edged sword as it can not only buffer stress and enhance life satisfaction, but can also increase both factors.

In previous studies, some researchers found that social media use promotes individuals' psychological well-being (e.g., happiness and satisfaction with life), however, more scholars found that social media use negatively affects their physical and mental health (e.g., depression symptoms and life dissatisfaction) and is related to greater loneliness (O'Day and Heimberg, 2021). Verduyn's et al. (2017) suggested that whether positive or negative effects of social media use on well-being are found might depend on the specific types of social media use: active versus passive. In other words, the effect of social media use on well-being is positive in case one uses social media actively; and vice versa. Furthermore, Verduyn's et al. (2017) also proposed that active usage of social media might positively affect individuals' social capital and connectedness, which, in turn, positively affects their subjective well-being. In contradiction, passive usage of social media positively affects upward social comparison and envy, which, in turn, negatively affects subjective well-being. Hence, Verduyn, Ybarra, Rsibois, Jonides and Kross (2017) research implies that increased usage of WeChat due to self-quarantine might be interpreted as a passive type of social media use, which may exert a negative impact on well-being (i.e., higher levels of perceived stress and lower levels of life satisfaction) in a direct way, and in an indirect way, that is through social loneliness, as well. Our findings support their review outcomes and add to the empirical knowledge in this field.

Third, we found that lockdown WeChat use intensity positively moderates the relationship between lockdown social isolation and lockdown social loneliness, which, in turn, partially mediates the relationship between lockdown social isolation and lockdown stress, and fully mediates the relationship with lockdown life satisfaction. These outcomes are important as during the COVID-19 lockdown many Chinese people's WeChat use intensity increased, herewith endangering their well-being (i.e., higher levels of lockdown stress and lower levels of life satisfaction). These findings also indicate that online social interactions, which presumably are valued by many people who believe that these can compensate for the lack of offline social interactions, relieve their perceived stress (anxiety) in actual life, and make their lives better (Kantar Group, 2018), are in fact endangering their mental health and life satisfaction during the COVID-19 lockdown period.

Hawkley and Cacioppo (2010) considered that the more lonely an individual feels, the more likely they develop negative attributions to others, which, in turn, lead to negative behaviors and a decrease in feelings of belonging and security. O'Day and Heimberg (2021) suggested that individuals who report a higher amount of loneliness are also more hungry for online social contacts and the more negatively compare themselves with others, which, in turn, may prevent them from experiencing the benefits of social media use, and drive them to use social media more passively. Accordingly, the more frequently or intensely a lonely person uses social media, the more lonely he or she feels, which, in turn, results in higher levels of stress and lower levels of life satisfaction. In addition, Putnam (2000) also argued that the amount of time spent on the Internet might crowd out time spent in socializing.

Analogously, for many Chinese people, increased use of WeChat may crowd out their time spent in offline social interactions, which, in turn, may lead to higher levels of stress and lower levels of life satisfaction. Indeed, all in all, our findings suggest that high levels of WeChat use intensity, which mainly originate from acute self-quarantine during the COVID-19 lockdown period in China, and which are believed to help people to cope with such a situation of forced social isolation and to relieve their anxiety and other mental symptoms (Zhang et al., 2020), rather strengthen the harmful effect of loneliness on their well-being instead of protecting them.

### Limitations and Suggestions for Future Research

Although our findings serve as a useful baseline for further investigations on the impact of WeChat use intensity on social isolation, loneliness, and well-being during the COVID-19 pandemic in China, the present study also has several limitations. First, this empirical work was based on respondents' self-reporting after the full lockdown was abolished, and this might lead to selection bias and memory bias. Moreover, there might be some concern whether common-method bias may have affected our results (Podsakoff et al., 2003). Fortunately, Harman's single factor test indicated that this was not a large problem in this study (ibid.).

Second, in China, social relationships and social structures are built on the hierarchical structure of family, and social relationships are often an extension of family relationships. As Fei xiao-tong once said: “The structure of Chinese society is like ripples caused by throwing a stone into a pond. Everybody is situated at the center of water rings, which are extended to reach an edge of one's social influence. No matter when and where one finds oneself, one is always situated at the center of the flexible social network” (Hwang, 1999, p. 173). In this context, social loneliness may be considered to be the reflection of emotional loneliness (which mainly originates from the lack of “family attachment”). Therefore, we did not examine the influence of WeChat use intensity on emotional loneliness.

However, as China is undergoing a drastic social transition, Chinese nuclear families are decreasing and single-person households are increasing (Peng and Hu, 2015). As a result, the meanings of social loneliness and emotional loneliness may have evolved considerably with the transition of family structure. Correspondingly, to get a more in-depth picture of the effect of WeChat use on well-being, future research is needed wherein a broader operarationalization of the concept of loneliness is used. Besides, WeChat use, stress, loneliness, and well-being may vary between countries, and therefore further evidence is needed to confirm the generalizability of the results across countries.

Moreover, future research is needed to further explore the model relationships using longitudinal approaches. More specifically, more insight into the stability and change of model variables and cross-relationships (i.e., overtime) can be provided by multiple-wave research (Taris and Kompier, 2003; De Lange et al., 2004). Specifically, from the perspective of social learning (Bandura, 2014), psychological functioning is dependent upon the continuous interaction among people, environment and behavior. Half of the prevalence of loneliness can be explained by heredity and half by one's environment (Boomsma et al., 2005).

Last but not least, some scholars have also found that high levels of loneliness might drive individuals to use WeChat and Internet more frequently in order to extend their interpersonal relationships online (Jiao, 2016) to compensate for their social isolation, that stress is associated with more hedonic and less eudaimonic media use (Eden et al., 2020), and that excessive use of social media during this period of isolation partially mediates the relationship between loneliness and anxiety (Boursier et al., 2020). Therefore, ideally, follow-up research is based on tracking data (e.g., China Health and Retirement Longitudinal Study, CHARLS) rather than cross-sectional data. If scientists in this field manage to collect more multi-wave data, a better understanding of the causality in our research model can be gained.

### Practical Implications

First, given the fact that high levels of WeChat use intensity may exert a more pernicious effect on stress and life satisfaction, through both direct and indirect ways, it is very important that self-isolated individuals prevent themselves from an excessive use of WeChat and Internet. In other words, individuals should prescribe themselves a limited time to access WeChat and Internet every day, and they should find beneficial substitutes (e.g., reading, exercising, playing games with family members), even in very difficult periods as during self-quarantine. In order to avoid people from the excessive and wrong use of social media like WeChat and the Internet, one's family, and representatives from school, government, enterprises and society should provide effective education for people to reduce the harm of over-use of social media. In particular, some small programs can be developed to help people to limit their online time. Once they overuse social media and the Internet, these small programs are meant to send warning messages to them.

Second, China is a society that is highly family-oriented (Yang and Yeh, 2008), and family has been an important source of economic strength and security for Chinese people since ancient times. High quality care and family companionship are good medicines against emotional loneliness, as the latter mainly results from the lack of “family attachment.” Hence, for Chinese people, instead of escaping in excessive WeChat and Internet use, it is important to establish and maintain good family relationships as these might reduce stress and anxiety, and enhance their satisfaction with life. Indeed, in China, family has become the most fundamental unit to cope with the adverse influences of both social isolation and loneliness, and to promote well-being, especially during the self-quarantined period due to the COVID-19 lockdown.

## Data Availability Statement

The data presented in this study are available on request from the corresponding author. The data are not publicly available due to privacy or ethical considerations.

## Ethics Statement

Ethical review and approval was not required for the study on human participants in accordance with the local legislation and institutional requirements. The patients/participants provided their written informed consent to participate in this study. Written informed consent was obtained from the individual(s) for the publication of any potentially identifiable images or data included in this article.

## Author Contributions

LZ: data curation and formal analysis. HT, SL, LZ, and ZG: funding acquisition. LZ, SL, and JL: investigation. LZ and JL: methodology. LZ and ZG: project administration. BV, ZG, and JL: supervision. All authors conceptualization, writing original draft, review and editing, read, and agreed to the published version of the manuscript.

## Conflict of Interest

The authors declare that the research was conducted in the absence of any commercial or financial relationships that could be construed as a potential conflict of interest.

## Publisher's Note

All claims expressed in this article are solely those of the authors and do not necessarily represent those of their affiliated organizations, or those of the publisher, the editors and the reviewers. Any product that may be evaluated in this article, or claim that may be made by its manufacturer, is not guaranteed or endorsed by the publisher.

## APPENDIX 1

**Table A1:** Description of measurement scales.

**Scale items (1 = strongly disagree, 5 = strongly agree)**	**References**
***Lockdown social isolation*****(*****CR*****=****0.829**, ***α*****=****0.825, AVE****=****0.621)**	
♢ I hadn't seen many of my family members whom I should have seen if there had been no lockdown. ♢ I hadn't seen many of my relatives whom I should have seen if there had been no lockdown. ♢ I hadn't seen many of my friends whom I should have seen if there had been no lockdown. ♢ I hadn't participated many activities which I should have been in if there had been no lockdown.	Ellison and George (1994); Brummett et al. (2001); Thoits and Hewitt (2001); Benjamins (2004); Cornwell and Waite (2009b); Zavaleta et al. (2017)
***Lockdown Loneliness(CR****=****0.847**, **α****=****0.846, AVE****=****0.649)***	
During the COVID-19 lockdown, ♢ I often felt that I lacked companionship. ♢ I often felt that nobody could truly understand me. ♢ I often felt left out. ♢ I often felt that I lacked having someone I could be close to. ♢ I often had feelings of being isolated and lonely.	Hughes et al. (2004); Schrempft et al. (2019)
***Lockdown stress*****(CR****=0.918**, **α****=0.917, AVE****=0.693)**	
During the period of COVID-2019 enclosed management, ♢ I often felt nervous and anxious. ♢ I tended to over-react to situations. ♢ I felt I was rather touchy. ♢ I found it difficult to relax. ♢ I found it hard to wind down.	Antony et al. (1998); Lee et al. (2019)
***Lockdown life satisfaction*****(*****CR*****=****0.831**, ***α*****=****0.823**, ***AVE*****=****0.624)**	
During the period of COVID-2019 enclosed management, ♢ My living conditions were quite good. ♢ My living standard was higher than that of other people around me. ♢ I was satisfied with how my life had gone. ♢ On the whole, I was satisfied with my life at that time.	Diener et al. (1985); Margolis et al. (2019)
***Lockdown WeChat use intensity*****(CR****=****0.888**, ***α*****=****0.884, AVE****=****0.726)**	
During the period of COVID-19 enclosed management, ♢ WeChat was a part of my everyday activity. ♢ WeChat was like a life companion. ♢ WeChat had become an indispensable part of my daily routine. ♢ I felt out of touch when I had not logged on to WeChat for a while.	Ellison et al. (2007); Moqbel et al. (2013); Wang et al. (2019)

*The values of CR, α, and AVE were calculated within the R packages “Lavaan” (Rosseel, 2012) and “SEMTools” (Jorgensen et al., 2021)*.
